# Caveolae Act as Membrane Reserves Which Limit Mechanosensitive *I*_Cl,swell_ Channel Activation during Swelling in the Rat Ventricular Myocyte

**DOI:** 10.1371/journal.pone.0008312

**Published:** 2009-12-14

**Authors:** Lukasz Kozera, Ed White, Sarah Calaghan

**Affiliations:** Institute of Membrane and Systems Biology, University of Leeds, Leeds, United Kingdom; Universidade Federal do Rio de Janeiro (UFRJ), Brazil

## Abstract

**Background:**

Many ion channels are preferentially located in caveolae where compartmentalisation/scaffolding with signal transduction components regulates their activity. Channels that are mechanosensitive may be additionally dependent on caveolar control of the mechanical state of the membrane. Here we test which mechanism underlies caveolar-regulation of the mechanosensitive *I*_Cl,swell_ channel in the adult cardiac myocyte.

**Methodology/Principal Findings:**

Rat ventricular myocytes were exposed to solution of 0.02 tonicity (T; until lysis), 0.64T for 10–15 min (swelling), and/or methyl-β-cyclodextrin (MBCD; to disrupt caveolae). MBCD and 0.64T swelling reduced the number of caveolae visualised by electron microscopy by 75 and 50% respectively. MBCD stimulated translocation of caveolin 3 from caveolae-enriched buoyant membrane fractions, but both caveolin 1 and 3 remained in buoyant fractions after swelling. *I*_Cl,swell_ inhibition in control cells decreased time to half-maximal volume (*t*_0.5,vol_; 0.64T), consistent with a role for *I*_Cl,swell_ in volume regulation. MBCD-treated cells showed reduced time to lysis (0.02T) and *t*_0.5,vol_ (0.64T) compared with controls. The negative inotropic response to swelling (an index of *I*_Cl,swell_ activation) was enhanced by MBCD.

**Conclusions/Significance:**

These data show that disrupting caveolae removes essential membrane reserves, which speeds swelling in hyposmotic conditions, and thereby promotes activation of *I*_Cl,swell_. They illustrate a general principle whereby caveolae as a membrane reserve limit increases in membrane tension during stretch/swelling thereby restricting mechanosensitive channel activation.

## Introduction

Caveolae are small (50–100 nm) invaginations of the plasma membrane, found in almost all cells of the body. They represent a specialised form of lipid raft, characterised by the presence of the small protein caveolin, which inserts into the inner leaflet of the membrane via a hairpin loop [1]. The assymetrical insertion of caveolin, and its tendency to cluster into oligomers gives caveolae their typical flask-like shape [2], [3]. Caveolin (Cav) is expressed as 3 major isoforms: Cav 1 and 2 (ubiquitously expressed) and Cav 3 (muscle-specific).

Caveolae have been shown to play a role in a variety of cellular processes including endocytosis, cholesterol homeostasis and signal transduction. Caveolae act as a compartment which brings together elements of the endocytotic machinery, lipid transporters and signal cascade components. Within caveolae, Cav acts as a regulator of protein activity and as a scaffold by interaction via its 20 residue scaffolding domain [4], [5]. In addition to their ability to compartmentalise signalling, another property of caveolae, as a reserve of extra membrane [6], may be relevant to their functional role.

We are interested in the contribution of caveolae to volume regulation during swelling in the cardiac myocyte. In the heart, cell swelling can occur during episodes of ischaemia and reperfusion. During ischaemia, metabolites such as lactate accumulate within the cell causing swelling which is exacerbated on reperfusion, when the hyperosmotic extracellular solution is exchanged for one with normal osmolarity. During swelling, ion channels are activated that regulate cell volume; the main volume-regulatory channel is the swelling activated chloride channel *I*_Cl,swell_ [7], [8]. During swelling, *I*_Cl,swell_ is considered to be acting as a mechanosensor responding to changes in membrane tension, rather than to decreased intracellular osmolarity, because it can also be activated in response to other mechanical stimuli [9], [10].

The first direct evidence for a link between caveolae and volume regulation was made by Trouet *et al.* [11]. These workers showed that Caco-2 cells, which do not express Cav, generate minimal membrane current in response to hypo-osmotic challenge, yet show a pronounced membrane current characteristic of *I*_Cl,swell_ following transfection with Cav 1. This group went on to show that transfection of Cav-expressing endothelial cells with truncated Cav 1 (Δ1–81), which displaces endogenous Cav 1 from the lipid raft membrane fractions, impaired activation of *I*_Cl,swell_ during swelling [12]. These data suggest that the *I*_Cl,swell_ channel or elements that regulate *I*_Cl,swell_ require caveolae/caveolin. Consistent with caveolae's role as a compartment, it has been suggested that they affect volume regulation by bringing together the *I*_Cl,swell_ channel with tyrosine kinases which regulate channel activity [12], [13]. In the cardiac myocyte this might be achieved through the clamping of inhibitory src kinases in an inactive configuration by Cav [14]–[17].

However, caveolae's property as a membrane reserve is also likely to be relevant to processes like volume regulation that depend on changes in membrane tension. The lipid bilayer can only increase in area by around 3% before rupture, so membrane reserves are essential to allow changes in cell volume to occur without irreversible cell damage [18], [19]. Sarcolemmal folds at the Z line and caveolae act as sources of available surface membrane in the ventricular myocyte [20], [21]. It has been estimated that 22% of the membrane is in caveolae in the adult rat ventricular myocyte [22], and stretch- and swelling-induced incorporation of caveolae into surface membrane has been reported in a preliminary study by Kohl *et al.* [21]. As a membrane reserve, caveolae could affect the activation of mechanosensitive channels that regulate cell volume (like *I*_Cl,swell_) by limiting increases in membrane tension for a given change in cell volume. However, the work of Trouet *et al.* [11] is not consistent with a role for caveolae as a membrane reserve which would limit, rather than enhance, *I*_Cl,swell_ activation.

The aim of the present study was to investigate the effect of swelling on caveolar morphology and density, and to determine the consequences of removing caveolae for processes that depend on *I*_Cl,swell_ activation (volume regulation, contractility) in the adult ventricular myocyte. Our data are consistent with the hypothesis that caveolae as a membrane reserve attenuate *I*_Cl,swell_ activation by limiting increases in membrane tension during swelling.

## Results

**Figure 1:**
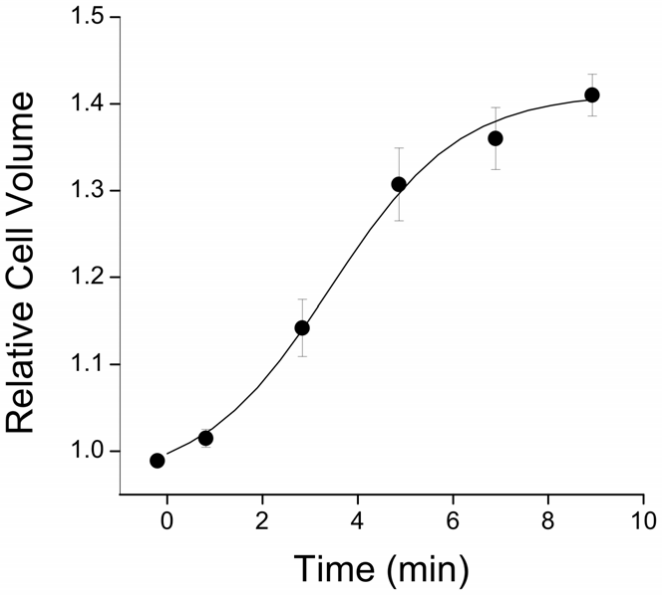
Time-course of myocyte swelling in response to 0.64T hypotonic solution. Myoycte volume was estimated from a video image of the cell, assuming the cell is an elliptical cylinder. Data are fitted with a logistic sigmoidal curve from which values of maximum cell volume and time to half-maximal volume were obtained. Mean±S.E.M. from *n* = 16 cells.

Figure 1 shows the change in myocyte volume in response to 0.64T hypotonic solution. After 9 min in hypotonic solution cell volume had increased by 41±2% with a time to half maximal volume of 3.4±0.3 min (*n* = 16 cells). Apparent surface area increased by 16±1% during this time. The time-course of changes in cell volume represents the sum of increased volume through osmotic swelling and decreased volume through volume-regulatory mechanisms, principally *I*_Cl,swell_. As volume and apparent surface area measurements are estimates based on a 2-dimensional image of the cell, we consider that it is the time-course of swelling that provides the most sensitive index of processes that modulate volume regulation.

### Effect of Hyposmotic Challenge on Caveolar Morphology

**Figure 2:**
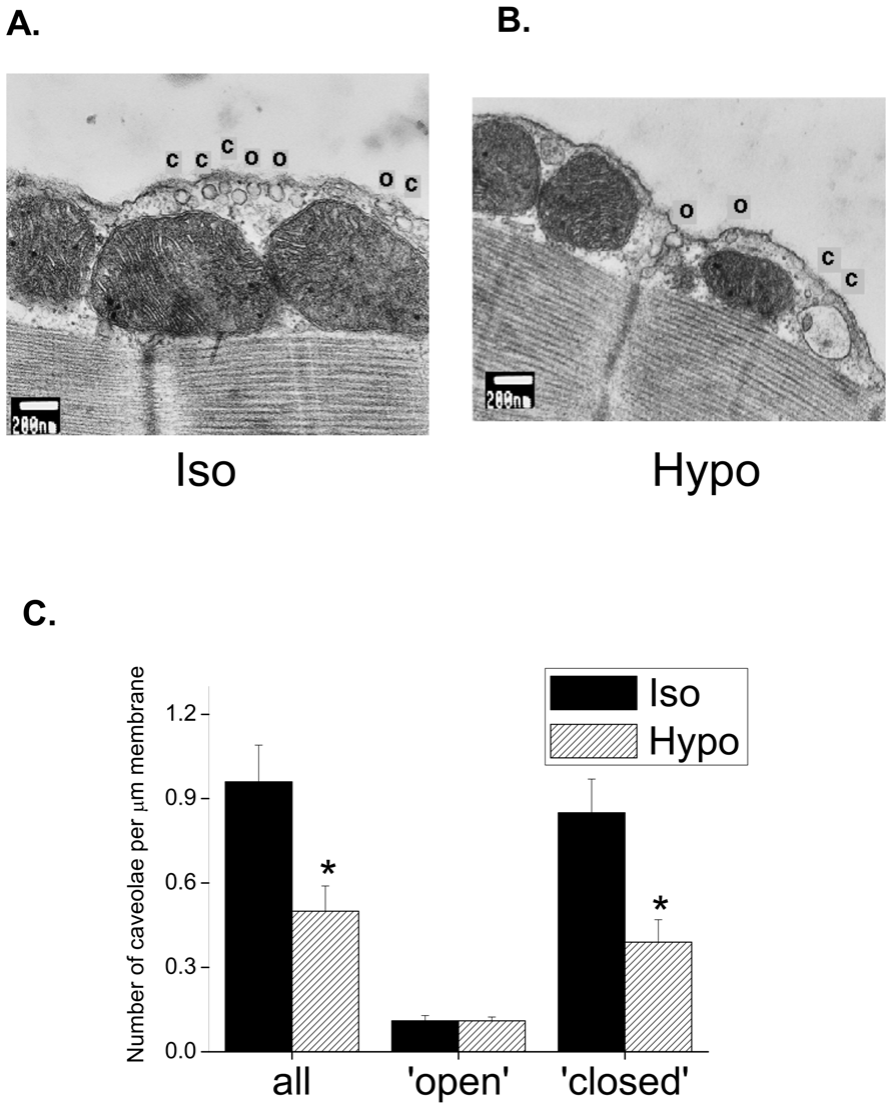
Hyposmotic swelling reduces the density of caveolae in the closed configuration. Ventricular myocytes were exposed to isotonic or hypotonic solutions for 15 min. Representative electron micrographs of membrane sections from myocytes in isotonic (A.) and hypotonic (B.) conditions. Caveolae were recorded in both open (O) and closed (C) configurations (see Results). C. Mean data showing that exposure to hypotonic solution reduced the total number of caveolae through a reduction in the closed, but not open, state (* P<0.05 unpaired Student's t-test, n = 9–11 cells).

Figure 2 shows representative electron micrographs of areas of cell membrane from myocytes exposed to isotonic and hypotonic solution for 15 min. In cells exposed to isotonic solution, both invaginations and closed subsarcolemmal vesicles are clearly evident with a diameter consistent with that of caveolae (50–100 nm). A proportion of apparently ‘closed’ caveolae could represent an artefact of sectioning within the bulb of the caveolae, but outside the neck (see Discussion). The density of caveolae was less in myocytes exposed to hypotonic solution (see Figure 2B). Figure 2C summarises mean data for the number of caveolae under isotonic and hypotonic conditions. Swelling was associated with a significant reduction (P<0.05) in the total number of caveolae by around 50%. When the 2 populations of caveolae were considered separately, it was clear that this reduction in caveolae number reflects a reduction in closed caveolae only; the number of open caveolae was identical under isotonic and hypotonic conditions.

### Disruption of Caveolae with MBCD

**Figure 3:**
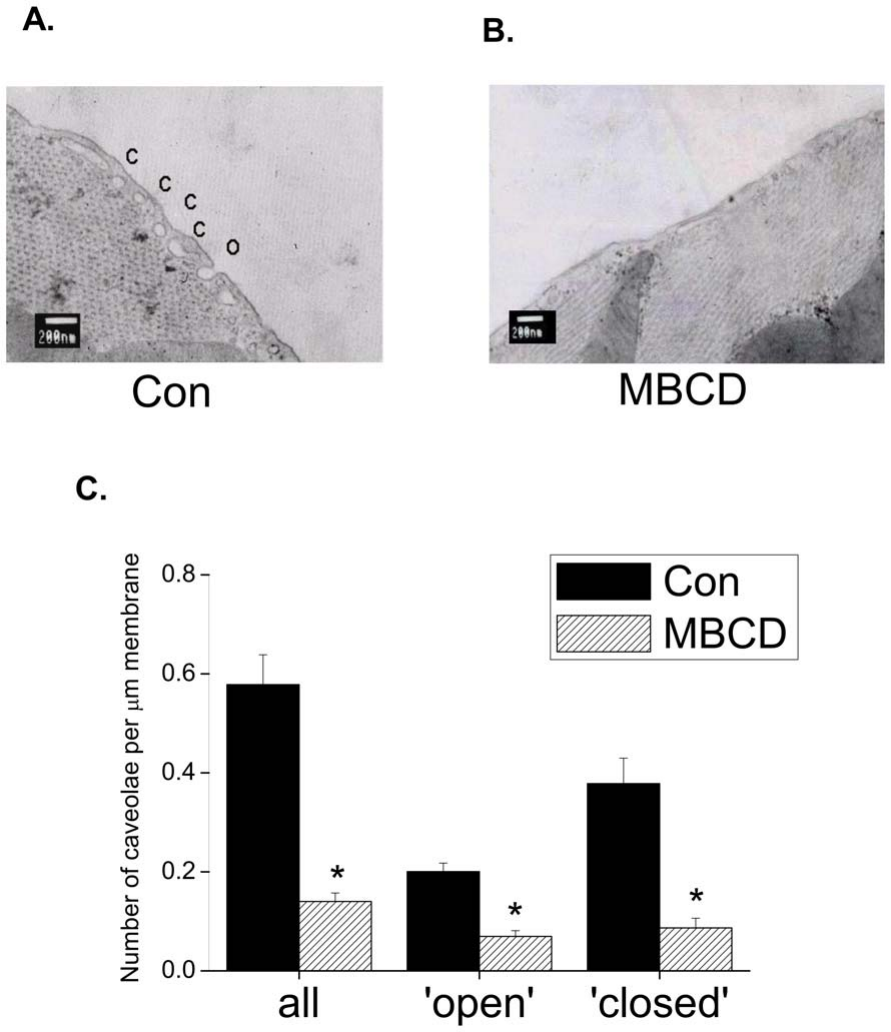
The cholesterol-depleting agent methyl β cyclodextrin (MBCD) reduces the density of open and closed caveolae. Representative electron micrographs of membrane sections from control (A.) and MBCD-treated (B.) myocytes. C. Mean data showing that MBCD reduced the total number of caveolae through a reduction in both open and closed configurations (* P<0.05 unpaired Student's t-test; *n* = 11 cells).

We have previously shown that treatment of myocytes with 2 mM MBCD for 1 h at 37°C depletes caveolae of their 2 essential components, cholesterol and Cav [23]. Here we show the effect of MBCD on caveolar morphology. Figure 3 shows electron micrographs of areas of cell membrane from control and MBCD-treated myocytes, and mean data for the number of open and closed populations of caveolae. The number of identifiable caveolae was reduced by 75% following MBCD treatment (P<0.05) which, by contrast to the effect of swelling, was due to a reduction in both open and closed populations (Figure 3C).

EM data from control cells (Fig. 3) allow us to estimate the proportion of membrane within the caveolar reserve in our myocytes. In ≈100 nm sections we see 0.6 caveolae per µm membrane (equating to 6 per µm^2^), which is identical to values reported in adult rat myocardium using transmission EM by Patel *et al.* [24], but lower than that reported by Gabella [22] (11 per µm^2^). The average diameter of a caveola is ≈75 nm which (assuming the caveola is a perfect sphere) means that the caveolar surface membrane area will be ≈0.02 µm^2^. Therefore, open and closed caveolae together contribute around 12% to membrane area in these cells. This is lower than a previous estimate of 22% [22], consistent with the difference in measured caveolar density (6 vs. 11 per µm^2^).

### Do Swelling-Induced Changes in Caveolar Morphology Cause Translocation of Cav 3?

**Figure 4:**
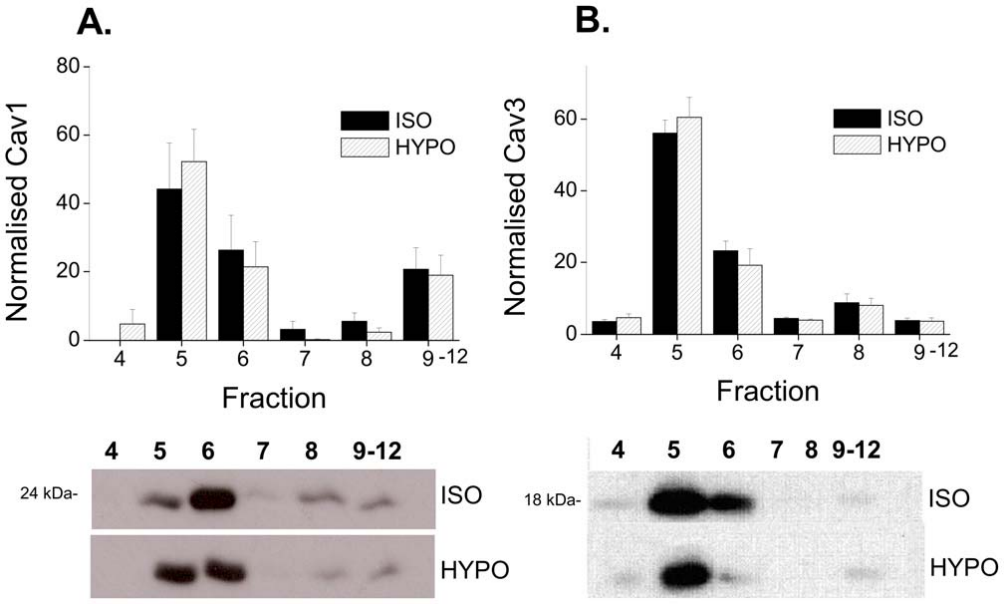
Hyposmotic swelling does not cause translocation of Cav 1 or Cav 3 from caveolar membrane fractions. Ventricular myocytes were exposed to isotonic or hypotonic solutions for 15 min, then subject to Na_2_CO_3_ extraction and discontinuous sucrose density gradient fractionation. A. Mean data showing Cav 1 in each fraction normalised to the sum of Cav 1 in all fractions for each sample (*n* = 6 hearts). Representative immunoblots, with equal volume loading of fractions, is shown below. B. Mean data showing Cav 3 in each fraction with representative immunoblots below. In both isotonic and hypotonic conditions, Cav 1 and 3 were enriched in the buoyant lipid raft fractions (5,6) of the myocyte. Hyposmotic swelling had no effect on the location of either Cav isoform.

Some mechanical stimuli cause translocation of Cav from caveolae which can have implications for signalling regulated by Cav [25]. Muscle-specific Cav 3 is the predominant isoform in the cardiac myocyte, however Cav 1 has recently been shown to be expressed in these cells [26], [27]. Figure 4 shows the distribution of Cav 1 and Cav 3 across membrane fractions under isotonic conditions and following 15 min exposure to hypotonic solution. In the cardiac myocyte, both isoforms of Cav are enriched in fractions 5 and 6 of the gradient which represent the buoyant cholesterol-containing lipid raft/caveolar fractions [23]. We saw no effect of swelling on the distribution of Cav 1 or Cav 3 between fractions; Cav remained predominantly in the buoyant membrane fractions. These data suggest that, although swelling reduces the number of caveolae, it does not stimulate translocation of Cav.

The molecular identity of *I*_Cl,swell_ is unknown, although several candidates have been proposed including C1C-3, P-glycoprotein and phospholemman (see [8]). Therefore, we were unable to show whether *I*_Cl,swell_ channels are present in the caveolae/lipid raft membrane fraction and whether swelling stimulates their translocation.

### Estimation of Surface Membrane and Intracellular Membrane Reserves

**Figure 5:**
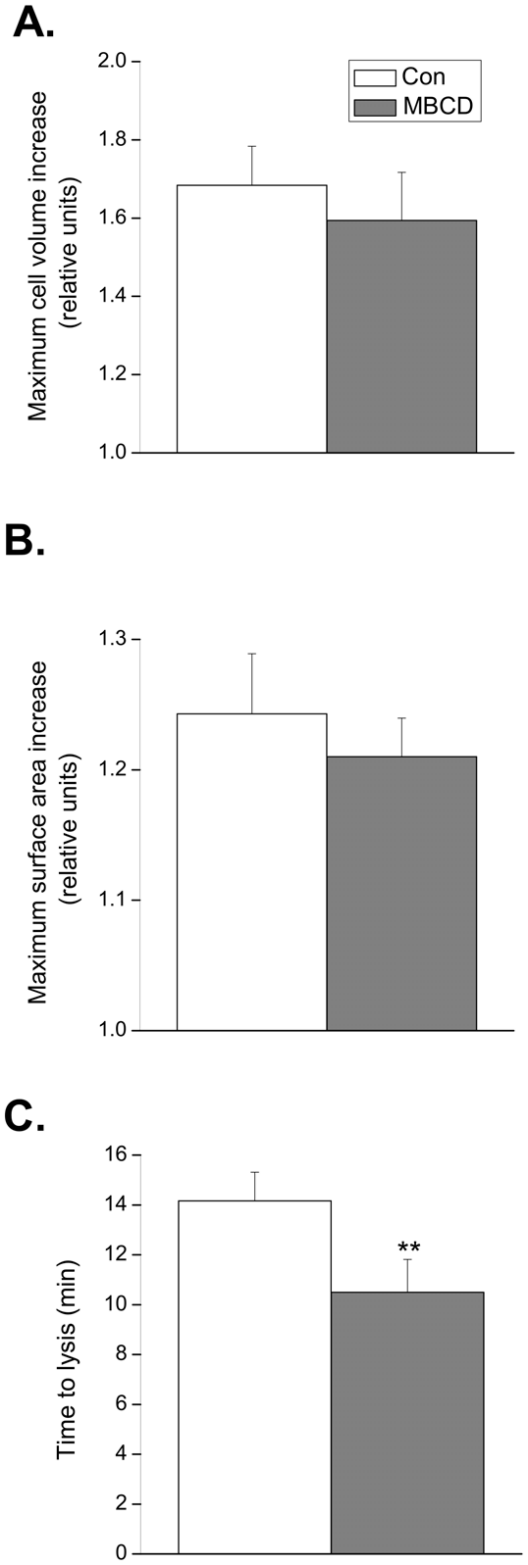
Effect of caveolar disruption on maximum cell volume, surface area and time to lysis in 5 mOsM solution. Quiescent ventricular myocytes were exposed to a 0.02T hypotonic solution and the maximum cell volume and surface area achieved before lysis was estimated from a video image of the cell. Disruption of caveolae with MBCD did not significantly alter the maximum cell volume (A.) or surface area (B.) prior to lysis. C. The time to lysis was significantly reduced in cells treated with MBCD (** P<0.01 versus control; Student's t-test; *n* = 11 cells).

Next, we measured the effect of disrupting caveolae with MBCD on the maximum increase in volume and surface area, and time to lysis in cells exposed to a solution of very low osmolarity (0.02T) in order to estimate the surface and intracellular membrane reserves (which include open and closed populations of caveolae respectively) [19]. These data are summarised in Figure 5. In control cells, the maximum increase in volume and apparent surface area were 68±10% and 24±5% respectively. There was a tendency for both parameters to be reduced in cells treated with MBCD, although these differences were not significantly different (P>0.05). However, disruption of caveolae significantly reduced (P<0.01) the time to cell lysis from 14.2±1.1 min in control cells to 10.5±1.3 min in MBCD treated cells. These data are consistent with caveolae acting as membrane reserves which protect the cardiac cell from lysis.

### Volume Regulation Following Inhibition of I_Cl,swell_ and/or Disruption of Caveolae

**Figure 6:**
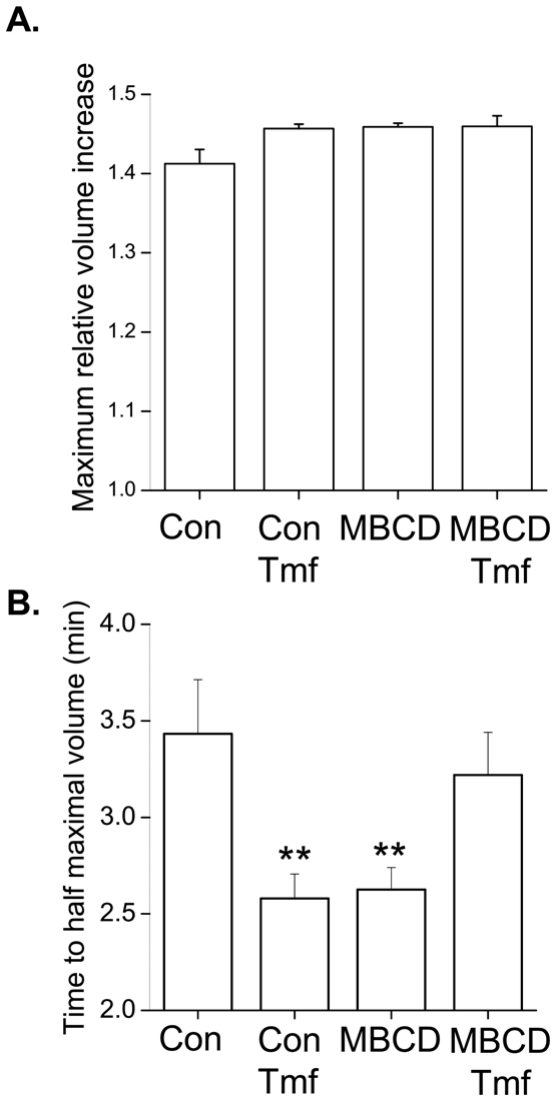
Comparison of the effect of caveolar disruption and *I*_Cl,swell_ inhibition on myocyte volume regulation. Values for maximum volume increase (A.) and time to half-maximum volume (B.) were obtained by fitting a sigmoidal relationship to the time-course of cell volume changes during 9 min exposure to 0.64T solution (see Figure 1). Inhibition of *I*_Cl,swell_ with 10 µM tamoxifen (Tmf) and/or disruption of caveolae with MBCD had no significant effect on the maximum volume achieved during swelling. By contrast, Tmf and MBCD both significantly reduced the time to half-maximal swelling, although the effects were not additive (** P<0.01 vs. control group (Con); ANOVA; *n* = 16–19 cells).

*I*_Cl,swell_ contributes to volume regulation in the cardiac myocyte, limiting cell swelling. In order to assess the way that caveolae regulate *I*_Cl,swell_, we compared the effect of *I*_Cl,swell_ inhibition and caveolar disruption with MBCD on the cell volume response to hyposmotic challenge (0.64T). In these experiments, tamoxifen (Tmf) was used to block *I*_Cl,swell_; Tmf is considered a relatively selective blocker of *I*_Cl,swell_ and at 10 µM fully blocks this current [28]. The time-course of swelling in control cells is shown in Figure 1. Figure 6 summarises values of maximum volume achieved during swelling and the time to half-maximal volume for control cells, cells in which *I*_Cl,swell_ was inhibited with tamoxifen, MBCD-treated cells, and MBCD treated cells in which *I*_Cl,swell_ was inhibited with tamoxifen. There was a trend for the maximal volume achieved during swelling to be higher following tamoxifen and/or MBCD treatment although this was not significant (P>0.05) (Fig. 6A). However both tamoxifen and MBCD significantly reduced the time to half-maximal swelling consistent with an increased rate of swelling (Fig. 6B). Tamoxifen treatment of cells in which caveolae were disrupted had no significant effect (P>0.05) on time to half-maximal swelling. Thus inhibition of *I*_Cl,swell_ and disruption of caveolae have identical (non-addditive) effects; both attenuate volume regulation in the cardiac cell.

### Effect of Caveolar Disruption on Shortening Response to Swelling

**Figure 7:**
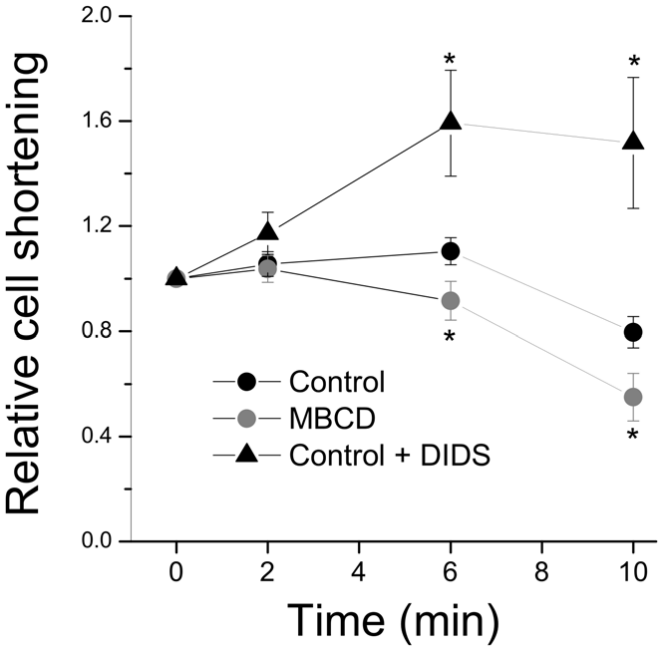
Effect of caveolar disruption and *I*_Cl,swell_ inhibition on the contractile response to hyposmotic swelling. Field-stimulated myocytes were exposed to 0.64T hypotonic solution for 10 min and the change in contractility (expressed as a % of resting cell length) measured. The negative inotropic response recorded at 6 and 10 min in control cells was enhanced in cells in which caveolae were disrupted with MBCD, and reversed in cells in which *I*_Cl,swell_ was inhibited with 50 µM DIDS. * P<0.05 vs time-paired control (ANOVA; *n* = 19–21 cells).

*I*_Cl,swell_ depolarises resting membrane potential and decreases action potential duration [7]. We have previously shown that 10 min exposure to hypotonic solution abbreviates the action potential duration in the rat ventricular myocyte, an effect sensitive to 4,4′diisothiocyanostilbene-2,2′-disulphonic acid (DIDS), confirming a contribution from *I*_Cl,swell_ [29]. The negative inotropic response observed after swelling has been ascribed in part to this *I*_Cl,swell_ -induced action potential abbreviation [29], [30]. We used the swelling-induced change in contractility as an index of *I*_Cl,swell_ to test how disrupting caveolae affects *I*_Cl,swell_ activation. As Tmf also blocks L type Ca^2+^ current [31], and because we observed a 70% reduction in contractility with 10 µM Tmf (data not shown), we used DIDS to inhibit *I*_Cl,swell_ in experiments involving measurement of cell contractility. In isotonic conditions, DIDS (50 µM) has no effect on action potential configuration [29] or basal cell contractility (present study; data not shown), confirming that it has no significant effect on currents flowing during the normal action potential in the absence of swelling. Figure 7 shows the effect of inhibiting *I*_Cl,swell_ and disrupting caveolae on the negative inotropic effect of swelling. *I*_Cl,swell_ inhibition with DIDS reversed the negative inotropic response to swelling, consistent with a role for *I*_Cl,swell_ in this. Although MBCD mimicked the effect of *I*_Cl,swell_ inhibition on the volume response to swelling (Fig. 6), in cells in which caveolae were disrupted with MBCD we saw a significant *potentiation* (P<0.05) of the negative inotropic response to swelling at 6 and 10 min after exposure to hypotonic solution relative to controls. This suggests that disrupting caveolae increases activation of *I*_Cl,swell_.

## Discussion

**Figure 8:**
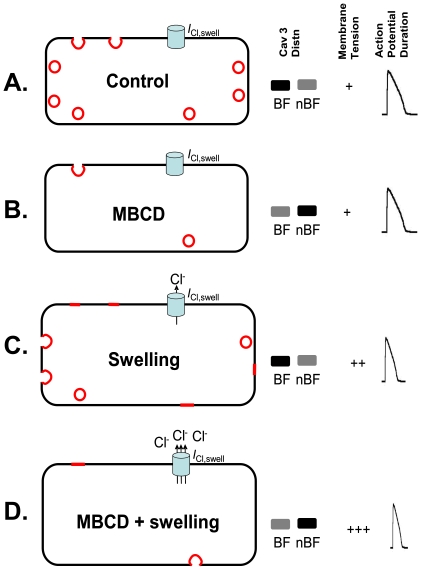
A simple model summarising the effect of cholesterol depletion and/or swelling on caveolae configuration, Cav 3 distribution, membrane tension and consequences for *I*_Cl,swell_ activation. A. Under control conditions, caveolae exist in open and closed configurations, Cav 3 is found predominantly in buoyant caveolae-containing membrane fractions (BF; fractions 5,6 of the sucrose gradient, see Fig. 4), and the *I*_Cl,swell_ channel is inactive. B. MBCD reduces the number of open and closed caveolae in association with translocation of Cav 3 from the BF to non-buoyant fractions (nBF; fractions 7–12), which contain heavy membranes and cytosolic proteins. The *I*_Cl,swell_ channel remains inactive. C. By contrast, swelling reduces the number of caveolae in the closed configuration only. We propose that this is through flattening of open caveolae in tandem with sarcolemmal incorporation of closed caveolae. No Cav 3 translocation from BF to nBF is seen during swelling. Membrane tension increases with swelling and this activates *I*_Cl,swell_ which abbreviates the action potential. D. In MBCD-treated cells, lack of available membrane reserves (open and closed caveolae) means that swelling increases membrane tension more than in control cells, causing enhanced *I*_Cl,swell_ activation, action potential shortening and negative inotropy. Cav 3-containing cholesterol-enriched membranes are shown in red: open caveolae (sarcolemmal red crescents); closed caveloae (intracellular red circles); flattened caveolae (sarcolemmal red lines). A representation of relative Cav 3 band density in BF and nBF, an index of membrane tension, and a representative action potential under each condition is shown to the right.

In this study we have looked at the effect of hyposmotic swelling on the morphology and Cav content of caveolae, and the impact of caveolar disruption on processes that depend on *I*_Cl,swell_ activation in the cardiac myocyte. We have shown that swelling has marked effects on the configuration of caveolae without affecting Cav localisation. Disrupting caveolae has significant effects on volume regulation and contractility in response to swelling, two processes which involve *I*_Cl,swell_ activation. Our data are consistent with the idea that disrupting caveolae removes essential membrane reserves so that cells swell more quickly, thereby potentiating activation of the mechanosensitive *I*_Cl,swell_ channel. A simple model summarising our findings is shown in Figure 8.

Using electron microscopy we have quantified for the first time the effect of hyposmotic swelling on the morphology and number of caveolae in the isolated cardiac myocyte. We see a 50% reduction in the number of caveolae (identified on the basis of size and shape) after 15 min exposure to hypotonic solution. This change in caveolae number reflects a decrease in the number of closed caveolae for which we cannot visualise a neck which connects with the sarcolemma. No change in the number of open caveolae was recorded. In agreement, Kohl *et al.* [21] have reported an absence of closed caveolae in rabbit myocardium after 30 min swelling with 0.75T solution.

In past decades there has been discussion as to whether closed subplasmalemmal vesicles of a size consistent with caveolae are truly closed caveolae, or whether they in fact represent a population of caveolae sectioned outside the connecting neck region. Early EM studies showed that in smooth, skeletal and cardiac muscle extracellular markers penetrate virtually all subplasmalemmal vesicles of a size consistent with caveolae, suggesting that they are relatively static structures open to the extracellular space [32], [33]. However, Gabella [22] reported nearly twice as many caveolae with transmission EM than with freeze fracture EM (which recognises only caveolae with connecting necks), suggesting that around half of caveolae are in the closed configuration. Moreover, increases in the number and diameter of connecting necks visible in freeze-fracture EM in response to increases in osmolarity have been reported in the atria [34], consistent with the idea that caveolar necks may be reversibly inserted into, and withdrawn from, the sarcolemma in response to changes in osmolarity. In the present study, on the basis of the neck:bulb diameter recorded in open caveolae (around 1∶2), a significant proportion of caveolae recorded in the closed state in control cells (85% of the total) should represent true closed vesicles. This is in accord with our observation that these closed caveolae are more distant from the sarcolemma than their open counterparts (see Fig. 2A).

We propose that the effect of swelling on reducing closed caveolae without affecting the open configuration underlies a cycle whereby stretch causes flattening of open caveolae in tandem with sarcolemmal incorporation of closed caveolae (see Figure 8). Kohl and co-workers [21] have previously reported evidence of stretch-incorporation of closed caveolae in the adult myocardium. The idea of increased membrane tension acting as a feedback mechanism for vesicle recruitment is well established [18], [35], [36].

Despite effects of swelling on the morphology of caveolae, we saw no translocation of Cav 1 or 3 from the caveolar fraction of the myocytes (see Figure 8). Whilst some have reported stretch-induced translocation of Cav 1 and 3 (in vascular smooth muscle) [25], this is not a universal mechanism. For example, although the small G proteins Rac and RhoA translocate from caveolae in axially stretch neonatal cardiac myocytes, Cav 3 was shown to remain in caveolae [37].

In the ventricular myocyte, the maximum volume and surface area increase recorded prior to lysis (68 and 24%) are much less than that reported for epithelial cells or fibroblasts (>1000 and 360%) [19], suggesting that the ventricular myocyte has less membrane reserves than these cell types. Groulx *et al.* [19] recently addressed the source of membrane reserves during modest and extreme swelling in cultured cell lines by measuring surface area and volume changes with or without functional exocytotic pathways. Their data suggest that, in epithelial cells and fibrobasts, the majority of membrane reserves required during modest swelling (<100% increase in cell volume) come from excess surface membrane rather than intracellular membrane stores (vesicles). In the present study, electron microscopy data suggest that, during exposure of the ventricular myocyte to 0.64T hypotonic solution (which gives a 40% increase in cell volume), membrane reserves come from both suface sarcolemma (open caveolae) and intracellular membrane (closed caveolae). We estimate that, in our myocytes, the extra surface area available from both open and closed caveolae is around 12%. This is less than the calculated increase in surface area during modest swelling (16%) and prior to lysis (24%), suggesting that other membrane reserves (e.g. Z-line folds, see [21]), are involved).

*I*_Cl,swell_ contributes to volume regulation in the cardiac myocyte. Here we have confirmed this by showing that the rate of swelling (indexed by time to half-maximal volume) in response to 0.64T solution is increased when *I*_Cl,swell_ is inhibited with tamoxifen. Disrupting caveolae with MBCD had an identical effect on the rate of swelling. We know that MBCD causes a translocation of Cav 3 from caveolae and a reduction in the number of identifiable caveolae [23], [24]. Therefore one interpretation of these data is that disrupting caveolae inhibits *I*_Cl,swell_, in other words, *I*_Cl,swell_ activation relies on caveolae (or the presence of Cav 3). This is in accord with work from Trouet *et al.* [11], [12] which suggests that caveolae act as a compartment to bring together the *I*_Cl,swell_ channel with elements necessary for its activation.

If *I*_Cl,swell_ activation relies on caveolae/Cav 3, then the negative inotropic response observed several minutes after swelling, which has been ascribed to action potential abbreviation secondary to *I*_Cl,swell_ activation [29], should be attenuated when caveolae are disrupted with MBCD. In contrast to this prediction, we saw that the negative inotropic response after 6 and 10 min of swelling was *enhanced* in cells treated with MBCD. These data are not consistent with the hypothesis that *I*_Cl,swell_ relies on caveolae/Cav 3, but suggest instead that disrupting caveolae promotes activation of *I*_Cl,swell_ by reducing the membrane reserves of the cardiac myocyte.

In this study we have shown, using EM and cell lysis, that disrupting caveolae removes essential membrane reserves. This means that cells in which caveolae are disrupted swell more quickly during exposure to 0.64T hypotonic solution. This effect can be mimicked by inhibiting *I*_Cl,swell_ which normally limits swelling during hyposmotic challenge. Because *I*_Cl,swell_ is activated more readily when there are less caveolae, we see a potentiation of the shortening response to swelling which reflects *I*_Cl,swell_-induced action potential abbreviation (see Fig. 8). Therefore, the present study presents a different mechanism for the role of caveolae in *I*_Cl,swell_ activation to that proposed by others in Cav 1-expressing cultured cells [11], [12]. We show that it is caveolae's role as a membrane reserve that is important in cell volume regulation in the adult cardiac myocyte.

The use of MBCD as an approach to selectively disrupt caveolae can have limitations because it also targets non-caveolar rafts (which are dependent on cholesterol). Furthermore, because MBCD affects both caveolar structure and Cav content (Fig. 3; [23]), MBCD does not distinguish between caveolae-dependent and Cav-dependent processes. However, the strength of the present study is that data cannot be explained by an effect of non-caveolar rafts or Cav on *I*_Cl,swell_ activity. If non-caveolar rafts or Cav keep *I*_Cl,swell_ inactive, then MBCD-treated cells would swell more slowly, which is opposite to the effect that we saw (Fig. 6). If non-caveolar rafts or Cav promote *I*_Cl,swell_ activation, then MBCD treated cells would swell more quickly (as we report) but the negative inotropic effect of swelling (which is proportional to *I*_Cl,swell_ activation) would be less (whereas we show that it is more; Fig. 7). So our data clearly show that it is the role of caveolae as a membrane reserve that predominates over all others in regulation of *I*_Cl,swell_ in the adult cardiac myocyte. We do not discount the possibility that the major role of caveolae as a membrane reserve may obscure more minor effects of non-caveolar rafts or Cav on *I*_Cl,swell_ in these cells.

This work is the first to show evidence for a role of caveolae in *I*_Cl,swell_ activation in the cardiac cell, and highlights a general mechanism by which caveolae could modulate mechanosensitive channel activation.

## Materials and Methods

### Ethics Statement

All animal experimentation was carried out in accordance with the Animals (Scientific Procedures) Act 1986 and conforms to the Recommendation from the Declaration of Helsinki and the Guiding Principles in the Care and Use of Animals. Animals were humanely killed by schedule 1 methods.

### Myocyte Isolation

Myocytes were isolated enzymatically from the hearts of male Wistar rats (250–280 g) according to the method described by Calaghan *et al.* [38]. Myocytes were treated with the cholesterol-depleting agent methyl-β-cyclodextrin (MBCD, 2 mM) for 37°C for 1 h to disrupt caveolae.

### Electron Microscopy

After treatment with isotonic or hypotonic solution for 15 min, a pellet of cells was formed by rapid centrifugation of the cell suspension at 80 *g*. Cell pellets were fixed overnight in 2.5% glutaraldehyde in isotonic or hypotonic solution as appropriate, post-fixed with 1% osmium tetroxide in 100 mM cocadylate buffer and dehydrated through an ethanol series. Cells were embedded in araldite and ultra thin sections (∼100 nm) were stained with 20% uranyl acetate (30 min) and Reynolds lead citrate (30 min) in the presence of 1 M NaOH. Sections were observed using a JEOL 1200 transmission electron microscope.

Caveolae were defined as flask-shaped invaginations (‘open’ caveolae) or subsarcolemmal vesicles (‘closed’ caveolae) with a size between ≈50 and 100 nm in diameter.

### Sucrose Density Fractionation

Preparations were fractionated using detergent-free methods as we have described previously [23]. Peripheral membrane proteins were extracted in 500 mM Na_2_CO_3_ (pH 11.0) containing 0.5 mM EDTA and 1% protease inhibitor cocktail (Sigma). Samples were homogenised (Ultra-Turrax T8; Ika), then sonicated (Vibra Cell; Sonics) 3 times each for 20 s at full power. Approximately 2 ml of homogenate was mixed with an equal volume of 90% sucrose in MES-buffered saline (25 mM MES, 150 mM NaCl, 2 mM EDTA, pH 6.5) to form a 45% sucrose solution. A discontinuous sucrose gradient was created by layering on to this a further 4 ml each of 35% and 5% sucrose solution (MES-buffered saline with 250 mM Na_2_CO_3_). Gradients were centrifuged for 17 h at 280,000 *g* (Beckman SW40Ti rotor) at 4°C. A total of 12 fractions (each 1 ml) were collected following fractionation. Cav 1 and Cav 3 were measured by Western blotting (antibodies 610406 and 610420 respectively; BD Biosciences) following SDS-PAGE [38]. Equal volumes of fractions were loaded onto the gel and the band density normalised to the sum of the band density in all fractions.

### Measurement of Cell Volume and Contraction

Isolated myocytes were placed in an experimental chamber on the stage of an inverted microscope and continually perfused with a HEPES-based solution (isotonic or hypotonic, see *Solutions*) at 22–24°C. For measurement of cell volume, cells were quiescent; for assessment of shortening they were field-stimulated at 0.5 Hz and cell length was monitored using an edge-detection system (Crescent Electronics). Cell volume was calculated from a video image of the cell using the relationship *V = (ΠwdL)/4* (where *V* is volume, *w* is width, *d* is depth and *L* is length, assuming the cell is an elliptical cylinder with the ratio of width to depth of 3∶1) [39]. Apparent surface area (which does not take into account any indendation/folds in the surface membrane) was estimated from *SA = 2ΠL (√((w/2)^2^+(w/6)^2^)*.

### Solutions

Isotonic physiological HEPES-based solution (Iso) contained (mM): NaCl, 57; HEPES, 5; Na_2_HPO_4_, 1; MgSO_4_ 7H_2_O 1, KCl, 5; sucrose, 113; glucose, 5.5; CaCl_2_, 1, pH 7.4 (280±10 mosM). Hypotonic solution (Hypo) had the same composition and pH as the isotonic standard physiological solution except that 113 mM of sucrose was omitted (180±10 mOsM; 0.64T). Cells were exposed to hypotonic solution for 10–15 min. For lysis experiments, hypotonic solution contained (mM): CaCl_2_, 1; MgCl_2_, 1. The pH was adjusted to 7.4 (5±2 mOsM; 0.02T).

For experiments to determine the effect of MBCD on caveolar morphology (Fig. 3), control physiological HEPES-based solution (Con) had the same composition as Iso but sucrose was omitted and [NaCl] was 137 mM.
